# Perioperative Management of Lithium Therapy: Considerations and Recommendations

**DOI:** 10.1111/bdi.70087

**Published:** 2026-02-05

**Authors:** L. S. Dominicus, F. van Houwelingen, M. Marsman, G. Lefeber, I. Wilting, A. Dols

**Affiliations:** ^1^ Department of Psychiatry University Medical Center Utrecht Utrecht the Netherlands; ^2^ Department of Anesthesiology University Medical Center Utrecht Utrecht the Netherlands; ^3^ Division of Internal Medicine and Dermatology, Geriatrics University Medical Center Utrecht Utrecht the Netherlands; ^4^ Department of Clinical Pharmacy University Medical Center Utrecht Utrecht the Netherlands

## 1 Lithium Safety and Monitoring

Lithium is a widely used mood stabilizer, primarily prescribed for patients with bipolar disorder (BD). It is also used as an adjunct therapy next to antidepressants in treatment‐resistant unipolar depressive disorder and, off‐label, as a prophylactic treatment for cluster headaches. Lithium has a narrow therapeutic index and its pharmacokinetics shows high inter‐ and intraindividual variation meaning that relatively small changes in serum level of lithium pose a significant risk of toxic or subtherapeutic serum levels, necessitating careful therapeutic drug monitoring. Lithium toxicity carries both acute and chronic health risks. In the short term, patients may experience neurotoxicity, which can manifest as confusion, cognitive impairment, and neuromuscular disturbances, alongside gastrointestinal symptoms and cardiovascular complications, and the rare but well‐described pulmonary manifestations of acute non‐cardiogenic pulmonary edema and acute respiratory distress syndrome (ARDS). Long‐term exposure to toxic lithium levels is linked to the development of progressive chronic kidney disease and cerebellar toxicity (ataxia and cognitive impairment), also known as syndrome of irreversible lithium effectuated neurotoxicity (SILENT). In recent years, lithium‐related renal decline has been linked to sustained supratherapeutic levels; this represents one of the risk factors for episodes of acute intoxication [1], urging clinicians to monitor levels carefully.

Monitoring lithium serum trough levels during the perioperative and postoperative periods is essential due to the risk of toxicity, which can result from fluctuations in lithium clearance due to changes in fluid/salt intake, hemodynamic changes caused by blood loss and/or fluid shifts, and the use of concomitant medications such as diuretics and NSAIDs. Cautious dosing is therefore recommended during these periods, with a preference for lower doses rather than higher ones. In clinical practice, the risk of manic or psychotic decompensation from subtherapeutic lithium levels for a few days is generally lower than the risk of acute lithium toxicity. Furthermore, acute psychiatric symptoms can be treated with rapid acting agents such as benzodiazepines and antipsychotics, whereas lithium toxicity may cause permanent renal and neurotoxic damage.

Clinical guidelines on perioperative management of lithium usually advise to discontinue lithium 24–72 h before major surgery [2, 3, 4], but lack specifying measures based on a low, intermediate, or high risk for intoxication.

Inadequate monitoring of lithium in the perioperative period poses significant risks, particularly when hemodynamic instability or impaired renal function affects sodium and potassium balance. Toxicity may develop rapidly, especially in patients with nephrogenic diabetes insipidus (NDI), a condition associated with large fluid loss which requires large fluid intake, affecting 20%–40% of long‐term lithium users. Symptoms of toxicity generally occur at serum levels above 1.5–2.0 mmol/L but may also arise at lower therapeutic levels, particularly in older patients. A key concern is that lithium toxicity may be mistaken for general illness or postoperative recovery, which can delay its recognition and increase the risk of developing chronic toxicity, potentially leading to irreversible cerebellar damage (SILENT).

**FIGURE 1:**
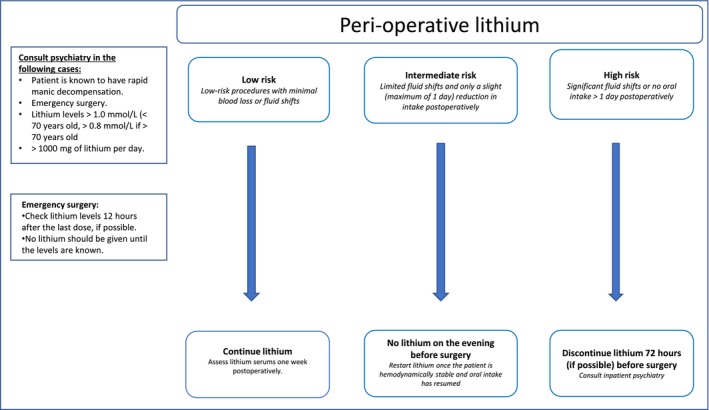
Flowchart for perioperative management of lithium.

In this paper, we present multidisciplinary recommendations based on clinical practice and current guidelines. These recommendations categorize procedures and patients into three categories (low, intermediate, or high risk for developing lithium intoxication) based on patient characteristics (i.e., age, comorbidity), fluid intake, the extent of surgery, and expected blood loss, in order to guide perioperative lithium monitoring (see Figure 1).

## 2 Perioperative Management of Lithium

### 2.1 Low Risk for Lithium Intoxication: No Expected Blood Loss, Fluid Shifts or Reduced Changes in Oral Intake Postoperatively

For patients undergoing procedures without anticipated significant blood loss or fluid shifts or reduced postoperative intake, lithium therapy can generally be continued without dose adjustments. This includes minor surgeries such as knee arthroscopy, hernia repair, breast surgery or ophthalmologic procedures. It is advised to the psychiatrist at the outpatient clinic of the patient to monitor lithium pre‐ and postoperatively. In cases when limited blood loss or moderate reduction in postoperative fluid intake is expected, the preoperative lithium serum level can be checked within the last few months before surgery by the treating psychiatrist, provided the treatment regimen is stable as long as clinically, and the patient has had no changes (e.g., renal function, drug–drug interactions) that increase intoxication risk; otherwise, in case of doubt, the test should be repeated 1–7 days before surgery. It is recommended to reassess the lithium serum level 1 week postoperatively, ensuring that levels remain in the target range.

### 2.2 Intermediate Risk for Lithium Intoxication: Limited Blood Loss or Fluid Shifts and Moderate Reduction in Postoperative Oral Intake

Example surgeries are total knee or hip repair or laparoscopic abdominal surgery. In cases when limited blood loss or moderate reduction in postoperative fluid intake is expected, the preoperative lithium serum level can be checked within the last few months before surgery by the treating psychiatrist, provided treatment regimen is stable, clinically, and the patient has had no changes (e.g., renal function, drug–drug interactions) that increase intoxication risk; otherwise, in case of doubt, the test should be repeated 1–7 days before surgery. Lithium should be stopped the evening prior to surgery and resumed 1 day after surgery, provided that patient remains hemodynamic stable, oral intake can be resumed, and renal function remains stable. It is advised to reassess lithium levels 1 week postoperatively.

### 2.3 High Risk for Lithium Intoxication: Significant Blood Loss, Fluid Shifts or Absence of Postoperative Intake

For surgeries involving significant expected blood loss or fluid shifts or no postoperative intake is assumed to be possible, lithium should be discontinued. Discontinuation is recommended 72 h before surgery (at least 24 h), given lithium's half‐life of 24–36 h. Reintroduction of lithium should only occur if potassium and sodium levels are normal, creatinine level is back to preoperative level, the patient is hemodynamically stable, and adequate fluid intake is provided. Lithium should be introduced gradually, and levels should be closely monitored upon reintroduction. The original dosage regimen can be resumed unless there is a decline in kidney function. In cases such as a cesarean section, dose adjustments may be necessary due to fluid shifts. Serum lithium levels should be evaluated 1 week after restarting lithium to ensure that therapeutic levels have been restored. For patients unable to take oral medications, lithium should be discontinued temporarily, as no parenteral substitution is available.

## 3 General Considerations

In addition, several general aspects are important for all patients.

In general, it is advised that patients alert clinicians that they are using lithium. Next, all lithium users should be offered written information about the use of lithium, its risks, signs of intoxication, and that specific medications can have an impact on their serum levels.

Prior to surgery, it is essential to gather clear information about the patient's lithium treatment, such as indication, time and dosing, and prescriber. Additionally, it is important to review the patient's target therapeutic lithium levels and the most recent measurements, while also assessing the individual's sensitivity to psychiatric destabilization due to fluctuations in lithium levels.

Furthermore, the presence of lithium‐induced NDI should be determined. If no formal diagnosis has been made, inquire about symptoms such as polydipsia or polyuria (defined as more than 3 L of fluid intake or urination per day), which may indicate possible NDI. If NDI is present or suspected, adequate urine monitoring should be performed and negative fluid balances should be corrected.

Next, additional laboratory tests should be assessed. If the patient's creatinine or GFR levels were measured more than 3–6 months ago, or if there is suspicion of declining renal function, these tests should be repeated. For high‐risk surgeries, creatinine and GFR must be measured within 1 month prior to the procedure. Electrolyte levels, including sodium and potassium, should also be evaluated unless results from the previous month are available. For intermediate‐ and high‐risk surgeries, electrolyte levels should be rechecked upon admission. However, testing thyroid function and calcium levels is only necessary if there are clinical signs suggesting (para)thyroid dysfunction. Regarding low and intermediate risks, routine preoperative lithium serum measurements are not required at the preanesthetic clinic, when a timed level arranged by the prescribing psychiatrist is already available. If indicated by clinical protocols, lithium serum levels should be measured 12 h (±30 min) after the last lithium dose. In BD, reference ranges for adults for therapeutic levels are 0.6–0.8 mmol/L for maintenance treatment and 0.8–1.2 mmol/L for treatment in acute mania or depression (age 60–79: 0.4–0.8 mmol/L and age 80+: 0.4–07 mmol/L [5]). For additional therapy in unipolar depressive disorder, reference ranges are 0.4–0.6 mmol/L. If a patient's psychiatric condition is stable, sometimes lower serum levels are accepted.

If lithium levels exceed 1.0 mmol/L in patients younger than 70, or 0.8 mmol/L in those older than 70, it is essential to consult a psychiatrist to determine whether the evening dose should be withheld. Postoperatively, lithium levels, creatinine, and electrolytes should be reassessed at day one, with further testing arranged in collaboration with psychiatry. Lithium serum levels should again be measured 12 h (±30 min) after the last dose.

For inpatients, pre‐ and postoperative monitoring white blood cell counts is necessary, as lithium may induce leukocytosis, which could be confused with postoperative inflammation. Special attention must be given to fluid intake, especially if the patient exhibits signs of polydipsia, as this may signal partial diabetes insipidus. Perioperative intravenous hydration should be carefully managed, adjusting for the patient's fluid balance.

Due to the variable risk of lithium toxicity, NSAID's should be avoided in the perioperative pain management.

## 4 Special Considerations

It is recommended to consult a psychiatrist if the patient has a documented history of rapid manic–psychotic decompensation in response to modest lithium serum level fluctuations. This information can be obtained by asking the patient or inferred from the medical history or prior correspondence. In the case of an emergency surgery, the advice is to discontinue lithium and check the lithium levels as soon as possible (by preference 12 h after last dose). Once the results are available, a psychiatrist should be consulted regarding the reintroduction of lithium or an alternative medication.

## 5 Conclusion

Lithium is most effective, but infamous for its renal side effects. Close monitoring of lithium serum levels around surgical procedures may prevent episodes of lithium toxicity, thereby limiting long‐term renal failure.

## Disclosure

All authors are part of a team responsible for the (protocols for) perioperative protocols and care for high‐risk surgical patients, both frail and with psychiatric disorders, with extensive clinical experience in psychopharmacology and managing complex cases and pharmacology.

## Data Availability

Data sharing is not applicable to this article as no new data were created or analyzed in this study.
